# A Change Talk Model for Abstinence Based on Web-Based Anonymous Gambler Chat Meeting Data by Using an Automatic Change Talk Classifier: Development Study

**DOI:** 10.2196/24088

**Published:** 2021-06-21

**Authors:** Kenji Yokotani

**Affiliations:** 1 Department of Clinical Psychology, Graduate School of Sciences and Technology for Innovation Tokushima University Tokushima-shi Japan

**Keywords:** problem gambling, web-based anonymous gambler chat meetings, self-help group, change talk classifier, computerized text analysis, long-term data with dropout gamblers, recovery gradient, gradient descent method, gambling, addiction, abstinence

## Abstract

**Background:**

Change and sustain talks (negative and positive comments) on gambling have been relevant for determining gamblers’ outcomes but they have not been used to clarify the abstinence process in anonymous gambler meetings.

**Objective:**

The aim of this study was to develop a change talk model for abstinence based on data extracted from web-based anonymous gambler chat meetings by using an automatic change talk classifier.

**Methods:**

This study used registry data from the internet. The author accessed web-based anonymous gambler chat meetings in Japan and sampled 1.63 million utterances (two-sentence texts) from 267 abstinent gamblers who have remained abstinent for at least three years and 1625 nonabstinent gamblers. The change talk classifier in this study automatically classified gamblers’ utterances into change and sustain talks.

**Results:**

Abstinent gamblers showed higher proportions of change talks and lower probability of sustain talks compared with nonabstinent gamblers. The change talk model for abstinence, involving change and sustain talks, classified abstinent and nonabstinent gamblers through the use of a support vector machine with a radial basis kernel function. The model also indicated individual evaluation scores for abstinence and the ideal proportion of change talks for all participants according to their previous utterances.

**Conclusions:**

Abstinence likelihood among gamblers can be increased by providing personalized evaluation values and indicating the optimal proportion of change talks. Moreover, this may help to prevent severe mental, social, and financial problems caused by the gambling disorder.

## Introduction

**Figure 1:**
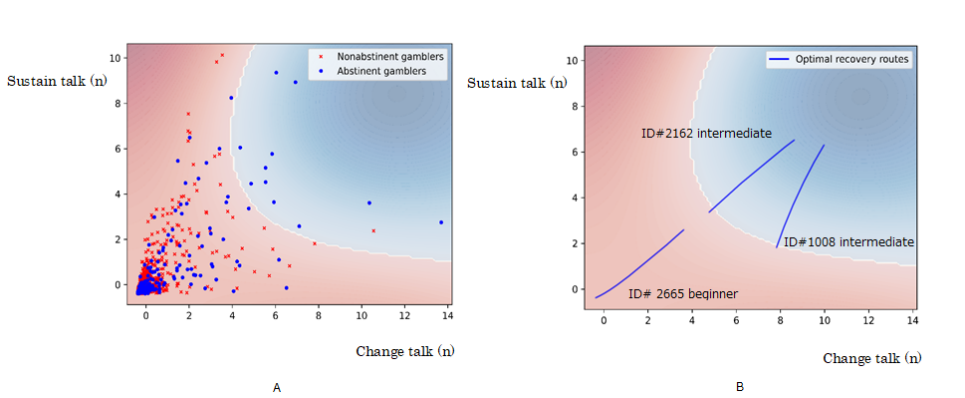
Change talk model for abstinence colored by evaluation values. Red areas indicate low abstinence likelihood areas (negative evaluation values), whereas blue areas indicate high abstinence likelihood areas (positive evaluation values). Horizontal and vertical lines represent standardized number of change and sustain talks, respectively. A: Scatter map of gamblers with and without current abstinent periods of at least three years. Blue circles and red Xs indicate abstinent and nonabstinent gamblers, respectively. B: Optimal recovery routes for 3 gamblers. Blue lines indicate 3 gamblers’ continuous optimal ways to increase their likelihood of gambling abstinence responding to their minute status differences. To clarify the meanings of the number of change and sustain talks, the numbers were unstandardized in the following cases. The beginner gambler (ID# 2665) started at 1 change talk and 1 sustain talk with evaluation value –1.000 and finished at 1657.16 change talks and 351.31 sustain talks with evaluation value –0.588 after 500 trials. The evaluation value at the end remained negative, thereby indicating low likelihood of gambling abstinence. The best portion of change talks (number of change talks/number of change and sustain talks) for the beginner (ID# 2665) during web-based anonymous gambler chat meetings was regarded as 0.8254. The intermediate gambler (ID# 1008) started at 3396 change talks and 259 sustain talks with evaluation value –0.063 and finished at 4290.42 change talks and 787.67 sustain talks with evaluation value +2.815 after 500 trials. The evaluation value at the end turned positive, thereby indicating high likelihood of abstinence. The best portion of change talks for the intermediate gambler (ID# 1008) during web-based anonymous gambler chat meetings was regarded as 0.6285.

Many individuals experience addictive disorders such as problematic drinking and gambling, and they require treatment to reduce the severity of their resulting mental and social problems [1,2]. One treatment option for such disorders is anonymous self-help group meetings, which have been proven to be as effective as standardized psychotherapies received in clinical settings [3]. Recent studies have shown that the abstinence process in anonymous self-help group meetings and standardized psychotherapies is similar [4], and the key factor in both treatments is to motivate participants to cease their addictive behaviors [5]. Studies have noted that individuals’ motivation can be measured by classifying their utterances during therapy at individual web-based [6] and offline group settings [7]. Further, their utterances can be automatically classified using a machine-learned classifier [8]. Thus, the abstinence process in web-based anonymous self-help group meetings can be clarified through machine-learned classification of utterances. However, the web-based abstinence process is unclear [5] despite its usefulness in terms of ease-of-use [9] and few damages caused by prejudice [10]. To clarify the abstinence process, a change talk model for abstinence was developed in this study based on the utterances of anonymous web-based gamblers through the use of a machine-learned classifier. The talk model developed in this study can be used to visualize the abstinence process of a gambler and specify his/her individual abstinence likelihood as well as the type of utterance that will encourage or prevent him/her from depending on his/her previous utterances (Figure 1).

The theoretical framework used in this study is the self-perception theory [11]. This theory assumes that individuals are persuaded by their own utterances and will thus behave in accordance with it. Individuals with drug addiction who continuously stated that they would not use drugs during therapy showed lower drug reuse after 1 year compared with those who did not make such statements [12]. Similarly, individuals with alcohol addiction who continuously stated that they would drink during therapy indicated higher alcohol reuse after 1 year compared with those who did not make such statements [13]. These findings indicate that individuals’ negative and positive comments on addictive behaviors were related to their improved and worse outcomes, respectively [14].

Based on these findings, motivational interviewing, a well-known method of motivating participants to cease addictive behaviors, classifies negative comments about addictive behaviors (eg, “Gambling is a waste of time”) and positive comments about ceasing addictive behaviors (eg, “I can save money because I stopped gambling”) together as “change talk” [14,15]. The results of a meta-analysis of motivational interviews showed that the proportion of participants’ change talks during therapy was linked with improved outcomes for addictive behaviors [16]. Studies have shown that abstinent drug users showed more change talks during therapy compared with nonabstinent drug users [17]. Similarly, abstinent gamblers also showed more change talks during therapy compared with nonabstinent gamblers [18]. These findings indicate that gamblers’ change talks are linked with their improved outcomes. Motivational interviewing also classifies positive comments about addictive behaviors (eg, “Gambling is the best game for me”) and negative comments about ceasing addictive behaviors (eg, “Since I stopped gambling, I've become more irritable”) together as “sustain talk” [14,19,20]. The results of a meta-analysis of motivational interviews showed that the number of participants’ sustain talks during therapy was linked with negative outcomes for addictive behaviors [16]. Another meta-analysis including dissertations also confirmed the relation of sustain talks with negative outcomes [21]. One more study showed that gamblers’ sustain talks were related with their negative outcomes [22]. These findings indicate that gamblers’ sustain talks are linked with their negative outcomes.

The change and sustain talk classifications were primarily utilized for face-to-face motivational interviewing therapy settings [16,21-23]; however, recent studies have applied the classification to other standardized individual therapies [24], group therapies [7], and web-based therapies [6]. These findings indicate that the classifications can be applied to web-based anonymous gambler chat meetings. Moreover, individuals with 3-year continuous abstinent periods were considered more robust abstinent individuals than those who had 90-day abstinent periods [25,26], although several studies have considered individuals with 90-day abstinent periods as abstinent individuals [3]. Several gambling studies have used data related to gamblers with 1-year continuous abstinent periods rather than those with 90-day abstinent periods [27,28]. In this study, gamblers with at least 3-year continuous abstinent periods were regarded as abstinent gamblers.

Based on studies on change and sustain talk classifications [16,21-23] and the change talk classifier [8], the change talk model in this study was developed to differentiate abstinent and nonabstinent gamblers. Recent studies have shown that instead of evaluating change and sustain talks separately, both must be evaluated simultaneously [16,22,23]. Hence, our study model considers the number of change talks and sustain talks as x and y axes variables in a scatter plot, respectively (Figure 1). Furthermore, the proportion of change talks (number of change talks/number of change and sustain talks) was evaluated in this study because studies have indicated that the proportion of change talks was a better index of improved outcomes than the number of change talks [16,22,23]. To validate this study’s findings with those of previous studies [16,22,23], this study hypothesized that the proportion of change talks among abstinent gamblers will be higher than that in nonabstinent gamblers in web-based anonymous gambler chat meetings.

## Methods

This study used registry data from the internet.

### Data Source

The author of this study accessed web-based anonymous gambler chat meeting data in Japan [29]. The meetings were conducted since September 2008 through web-based text chats with no pictures or sounds. A few offline meetings were conducted for a year in Osaka, Tokyo, or Sapporo. The web-based anonymous gambler chat meetings has no relationship with the Gamblers Anonymous group [28]. To post in the meetings, users had to be approved by the administrator in advance. Individuals who could participate in the meetings were those who were experiencing a gambling problem or their family members who were experiencing a gambling problem. The meetings were conducted by gamblers without any strict rules, and no outside experts were involved at all. The meetings were divided into those for users aiming for 1 week, 1 month, 3 months, 6 months, 1 year, 3 years, and more than 3 years of gambling abstinence, and users attended the meetings based on the number of days they had remained gambling-abstinent. However, these meetings had no strict rules, and a user aiming for 3-month gambling abstinence may attend a meeting for those aiming for 1-month gambling abstinence and vice versa. There were meetings for chatting as well as for providing daily reports and reports of memorial days (such as 100 abstinent days). Furthermore, these meetings were always open and free to join; therefore, the time and duration of participation varied widely among users (Table 1). Table 1 shows a comparison of the demographic variables, gambling history, gambling symptoms, participation forms, and characteristics of the utterances in web-based anonymous gambler chat meetings between abstinent and nonabstinent gamblers. In Table 1, abstinent gamblers refers to gamblers who have remained abstinent without relapse for at least three years. The utterance classifier in this study involves 6 clusters, but only participants’ change talk and sustain talk clusters were used in this study. The data size of the demographic variables and gambling histories was smaller than that of the utterances. The proportion of change talks was the number of change talks per the total number of change and sustain talks. Some users did not have any change and sustain talks; thus, their proportions were not calculated. When a single meeting exceeded 5000 posts, it would end and a new meeting would be started. These meetings spanned multiple days or months and were rarely completed within a day.

**Table 1:** Comparison of the demographic variables, gambling history, gambling symptoms, participation forms, and characteristics of the utterances in web-based anonymous gambler chat meetings between abstinent and nonabstinent gamblers.

Characteristics		Abstinent gamblers (n=267), mean (SD)	Nonabstinent gamblers (n=1625), mean (SD)	*d*	*t (df)*	*P* ** value
**Demographic variables**						
	Age (years)	35.000^a^ (9.520)	35.811^b^ (9.440)	–0.086	–0.896 (172.806)	.37
	Proportion of males	0.845^c^	0.860^d^	N/A^e^	–0.565 (256.025)	.57
**Gambling history**						
	Total amount of debt (million ¥)^f^	3.718^g^ (6.602)	2.159^h^ (3.727)	0.003	1.177 (27.320)	.25
	Length of gambling (years)	15.301^i^ (23.030)	11.843^j^ (7.350)	0.202	1.272 (74.526)	.21
**Gambling symptoms**						
	Number of symptoms (min:1, max:10)	2.981 (1.801)	2.661 (1.766)	0.179	2.696 (355.278)	.01
	Gambling tolerance	0.408 (0.492)	0.371 (0.483)	0.076	1.146 (355.382)	.25
	Unsuccessful control over gambling	0.730 (0.445)	0.733 (0.442)	–0.007	–0.104 (358.057)	.92
	Preoccupied with gambling	0.667 (0.472)	0.542 (0.498)	0.257	3.969 (370.285)	<.001
	Lies in gambling	0.311 (0.464)	0.198 (0.399)	0.261	3.750 (333.748)	<.001
	Reliance on others to provide money	0.094 (0.292)	0.079 (0.270)	0.051	0.747 (345.207)	.46
	Illegal acts for gambling	0.004 (0.061)	0.040 (0.196)	–0.250	–5.907 (1309.252)	<.001
**Participation forms in** **web-based** **anonymous gambler chat meetings**						
	Participation length (days)	842.974 (904.471)	386.072 (616.142)	0.590	7.957 (307.817)	<.001
	Interval length (days)	16.168 (63.090)	24.241 (104.712)	–0.093	–1.735 (543.061)	.08
**Utterance characteristics in** **web-based** **anonymous gambler chat meetings**						
	Number of total utterances	2395.625 (5146.820)	613.065 (1797.483)	0.462	5.603 (276.750)	<.001
	Number of change talks	410.007 (764.597)	115.869 (303.165)	0.506	6.206 (279.889)	<.001
	Number of sustain talks	98.944 (192.787)	37.585 (97.324)	0.402	5.095 (288.661)	<.001
	The proportion of change talks	0.742^k^ (0.200)	0.681^l^ (0.231)	0.280	4.477 (392.158)	<.001
	Average probability of change talks	0.298 (0.155)	0.299 (0.160)	–0.001	–0.022 (365.505)	.98
	Average probability of sustain talks	0.096 (0.062)	0.122 (0.076)	–0.369	–6.017 (406.651)	<.001
	The proportion of change talks’ probability	0.730 (0.150)	0.685 (0.167)	0.285	4.495 (382.728)	<.001

^a^n=129.

^b^n=771.

^c^n=193.

^d^n=1118.

^e^N/A: not applicable.

^f^¥1 million=US $9103.

^g^n=26.

^h^n=182.

^i^n=73.

^j^n=427.

^k^n=266.

^l^n=1612.

### Sample Size

Research focusing on 3-year continuous abstinent periods is limited [27]; therefore, this study used 3-year-continuous abstinent smokers’ studies [25] because smokers and gamblers share similar biological and environmental factors [30]. In the previous study [25], 13.8% of smokers achieved 3-year abstinence with the help of treatment whereas 86.2% did not. Based on the abovementioned findings [25], the allocation rate between abstinent and nonabstinent gamblers was set as 0.16. To show satisfactory power (0.95) with alpha (.05) for small effect size (*d*=0.2), 2736 participants were required.

### Participants

**Figure 2:**
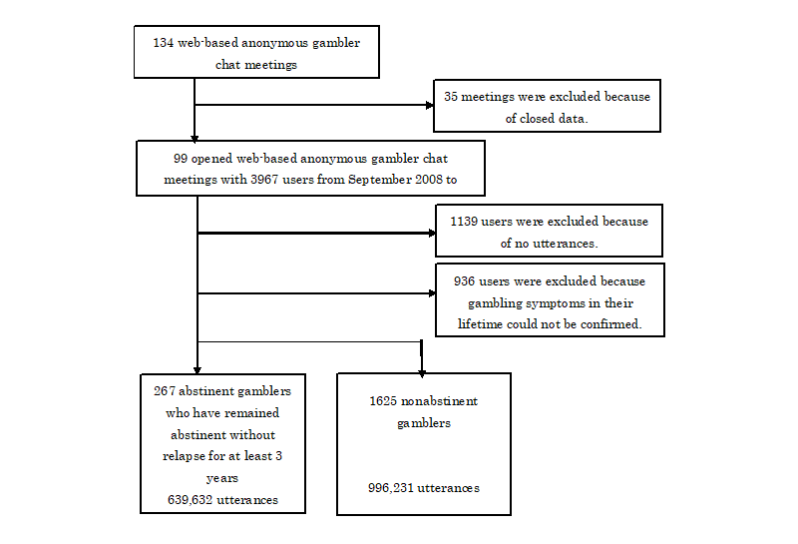
Data extraction of the participants.

A total of 134 web-based anonymous gambler chat meetings were web scraped on March 10, 2020 (Figure 2) [29]. Among them, 35 meetings included private data and were thus excluded. From the 99 remaining meetings, 3967 users were identified based on their anonymous names. They posted Japanese texts in the meetings from September 8, 2008 to March 10, 2020. Among them, 1139 gamblers were excluded because their posts included lesser than 3 words or included only advertisements with “http.” Furthermore, 936 users were excluded because we could not confirm the gambling symptoms in their lifetime based on their posts (the evaluation of gambling symptoms is presented in the next section). The final participants were 1892 gamblers who experienced at least one gambling symptom in their lifetime. The number of study participants (n=1892) did not reach the ideal sample size (n=2736). To show satisfactory power (0.95) with alpha (.05) in the 1892 participants, the effect size must be over 0.24.

### Collection of Participants’ Basic Data

#### Gambling Symptoms

Among the users’ posts, texts were extracted based on keywords related to 10 gambling symptoms, that is, 1 gambling symptom related to illegal behaviors in gambling [31] and 9 symptoms of gambling disorder as mentioned in the fifth edition of the American Psychiatric Association’s Diagnostic and Statistical Manual of Mental Disorders [1]. For example, “lie” is a keyword used for examining the symptom of lies in gambling; therefore, all texts involving “lie” were extracted. Two raters who majored in clinical psychology as undergraduate students and received 2-hour training regarding gambling disorders from a Japanese clinical psychologist [32] independently read the texts and evaluated whether the user had experienced the aforementioned 10 symptoms in their lifetime. To check the validity of their evaluation, the psychologist also blindly evaluated randomly selected texts from 100 participants. Kappa coefficients confirmed that the psychologist and the 2 raters were primarily in “almost perfect or perfect agreement” [33] in their evaluation of the presence of gambling symptoms (0.835 and 0.860, Table S1 of Multimedia Appendix 1). Based on their assessments, participants with at least one gambling symptom in their lifetime were included as the final participants (Figure 2). Furthermore, the number of gambling symptoms as well as the gambling symptoms for which agreement between the psychologist and the 2 raters was above “substantial agreement” (0.60) [33] are also listed in Table S1 (Multimedia Appendix 1) as the basic data for the user.

#### Demographic Variables

The sex and age of the participants were estimated using the first 1000 words of the aforementioned gamblers because these words frequently involved self-introduction statements. One-third of these gamblers were determined using a Japanese application [34] (Table 1).

#### Gambling History and Debt

Among the gamblers’ words, text was extracted based on the keywords, including “years” and “history,” and the text was read by the 2 raters. If the text revealed a gambling history, the user’s years of gambling were recorded (Table 1). Similarly, the text was extracted based on keywords such as “yen,” “ten thousand,” “cash,” or “borrow.” If the text indicated a debt, the debt was listed (Table 1). Missing data were excluded.

#### Participation Forms in Web-Based Anonymous Gambler Chat Meetings

The length of the participation in the meetings was estimated based on the days of the initial and final posts in the meetings. Furthermore, participants’ posts in the meetings involving long paragraphs were divided into 2 sentence units. If sentences were lesser than 3 words or included “http,” they were removed. A two-sentence text will herein be referred to as an utterance. The average interval of utterances was also estimated based on users’ participation length and number of utterances.

### Outcomes

Users who registered as gamblers were provided with a personal counter that automatically counts the number of days wherein they ceased gambling. If they gamble, they report it to the administrator who resets the number of days on the counter. Those whose counters exceed 3 years are listed on a separate website and exemplified as models who have stopped gambling. According to the list on March 2020, 267 gamblers were regarded as abstinent gamblers without relapse for at least 3 years with 639,632 utterances; the other 1625 gamblers were regarded as nonabstinent gamblers with 996,231 utterances. These counters and lists were created by the administrator without the involvement of the author.

### Implementation of the Change Talk Classifier

#### General Schema of the Change Talk Classifier

**Figure 3:**
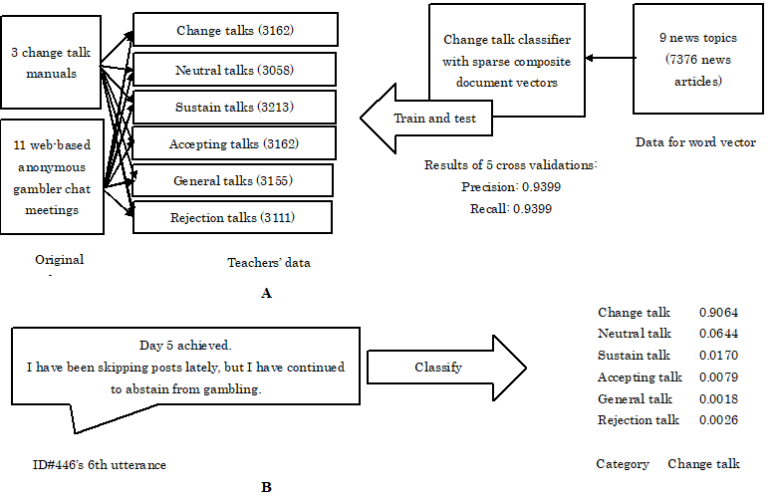
Implementation of an automatic change talk classifier. A: Data sets for the change talk classifier; B: An example of a change talk classifier.

To create a change talk classifier, sparse composite document vectors were used [35]. A total of 7376 Japanese news articles were used to create a 200-dimensional word vector [36] (Figure 3A). To construct the document vector, 18,861 utterances were used with 6 clusters: clients’ change, neutral, and sustain talks, as well as therapists’ accepting, general, and rejection talks [15,19,20] (Figure 3A). Although each cluster has subcategories, the utterances were classified into clusters instead of subcategories. Furthermore, half of the utterances in each cluster was derived from 3 change talk manuals [15,19,20]; the other half came from the initial parts of 11 web-based anonymous gambler chat meetings [29].

#### Creation of Teachers’ Data

Comments in the 3 manuals focused on other addictive behaviors [15,19,20] and were converted into gambling topics, for example, the comment “I am going to stop smoking tomorrow” was changed to “I am going to stop gambling tomorrow.” Additionally, several types of gambling are noted in Japan; therefore, the word “gambling” was used to replace 17 specific gambling terms such as “pachinko (Japanese pinball) game” and “horse racing.” In addition, the Japanese language includes several types of second personal pronouns [37]; therefore, 3 typical personal pronouns were used, namely, “anata,” “satosan,” and the zero pronoun. Each comment in the manual was converted into 51 different gambling comments (17 gamble types × 3 pronoun types). Finally, 31,977 utterances related to 6 clusters were obtained from the manuals. Furthermore, 9519 utterances were identified during the initial parts of the 11 web-based anonymous gambler chat meetings. These utterances were encoded by a Japanese clinical psychologist with expertise in mail-based motivational interviewing [38]. Finally, 9519 utterances in the meetings related to the 6 clusters were obtained.

#### Preprocessing of Teachers’ Data

Comments longer than 2 sentences were considered as an utterance. When the comment comprised a single sentence, the sentence was randomly combined with another comment from the same type of gambling (eg, “pachinko”), pronoun (eg, “anata”), and subcategory (eg, “ability in change talks”) utterances. The following 2 points were included as constraints. First, one cluster from one source should have 1500 utterances to reflect the rate of comments within the subcategories. Second, if the number of utterances in a subcategory was lesser than 51 because of the first constraint, the utterances should continue until they exceed 51. Finally, we obtained 18,861 utterances tied with 6 clusters. During the training and testing of the change talk classifier in this study, the number of words in each utterance was fixed at 200 to remove the effects of this aspect on the classification. For utterances of more than 200 words, the exceeding words were removed. For utterances lesser than 200 words, the 2 sentences were repeated until they reached 200 words.

#### Performance of the Change Talk Classifier

Among the 18,861 utterances in 6 clusters (Figure 3A), 4/5 were used as training data and 1/5 was used as test data. The training and test sessions were repeated 5 times. The average scores of precision and recall during these 5 sessions were 0.9399 and 0.9399, respectively. Utterances in web-based anonymous gambler chat meetings were labeled based on the classifier used in this study. For example, the classifier showed the estimated probability of the following utterance: “Day 5 achieved. Although I have been skipping posts lately, I have continued to abstain from gambling” (Figure 3B). The estimated probability of change talk was 0.9064, which is the maximum probability among the 6 clusters. Hence, the utterance was labeled as change talk (Figure 3B). Similarly, 1,635,863 utterances in the meetings were classified into 6 clusters. The classifier in this study did not involve the list of abstinent gamblers; the classifier was blind to the outcome.

### Independent Variables

#### Change Talk

Based on the change talk classifier, the number of change talks among 1892 gamblers was evaluated (Table 1). The estimated probability that the classifier uses for classification was utilized (Figure 3B) and showed the average probability of the change talk (Table 1).

#### Sustain Talk

Similarly, the number of sustain talks was evaluated (Table 1). The estimated probability for the classification was utilized (Figure 3B) and showed the average probability of the sustain talk (Table 1).

#### Proportion of Change Talks

Based on previous studies [16,22,23], the proportion of change talks (number of change talks/number of change and sustain talks) was evaluated in this study. Similarly, the proportion of change talk probabilities (average probability of change talks/average probabilities of change and sustain talks) was evaluated.

### Statistical Analysis

#### Model Comparison

Our change talk model for abstinence involved the number of change and sustain talks as independent variables and 3-year abstinence as outcomes (Figure 1). The number of change and sustain talks was high; therefore, they were standardized in the model (Figure 1). To find the best identification function for abstinent and nonabstinent gamblers, a support vector machine (SVM) was compared with a linear and (nonlinear) radial basis kernel function (RBF). The cost of penalty and complexity of boundary area parameters were estimated using the grid search method [39] from the set of 10^–4^, 10^–3^, 10^–2^, 10^–1^, 1, and 10^1^. The 3 cross-validation accuracies of SVM with RBF (0.8555, where cost of penalty and complexity of boundary area were 10^1^ and 10^–1^, respectively) outperformed the accuracy of SVM with linear function (0.8516, where cost of penalty and complexity of boundary area were 10^–3^ and 10^–3^, respectively). Hence, we used the SVM with RBF as the identification function for abstinent and nonabstinent gamblers. The SVM has an evaluation value that assesses the abstinence likelihood of gambling, with a negative score indicating low abstinence likelihood and a positive score indicating high abstinence likelihood (Figure 1).

#### Visualization of the Optimal Abstinence Process

To visualize the differences in the optimal abstinence processes of gamblers, the gradient descent method was used:

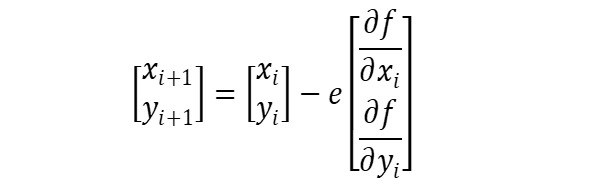



where *x_i_* and *y_i_* are the standardized number of change and sustain talks in trial *i*, respectively; *f* is the SVM identification function with RBF; 
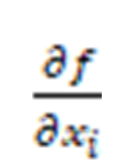
 and 
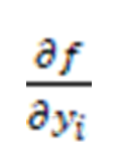
 are partial differentials of *f* at the point *x_i_* and *y_i_*, respectively; and *e* is the learning rate set as 0.01. The maximum number of trials was set to 500. Furthermore, the current *x_i_* and *y_i_* monotonically increase and the area of the partial differential is limited within the first quadrant. Moreover, when the score of *f* at trial *i* was better than that at trial *i* +1, the score of *f* at *i* was considered a local solution and the trials were ceased.

#### Software Used

For sample size estimate and power analysis, G*power 3.1.9.4 was used [40]. To determine users’ age and sex through text, COTOHA [34] was used. For Japanese text analysis, MeCab [41] was used. The original Python codes [35] were used to create the document vector.

### Ethical Considerations

This study was approved by the ethics committee of a National University in Japan (Reception number 222, ethical review on September 1, 2020). Furthermore, all procedures were conducted in accordance with the guidelines for studies involving human participants, the ethical standards of the institutional research committee, and the revised 1964 Helsinki Declaration and its later amendments or comparable ethical standards.

## Results

### Comparison of the Basic Characteristics Between Abstinent and Nonabstinent Gamblers

The gamblers examined in this study were primarily males (1125/1311, 85.8%) and aged around 35 years (Table 1). Although their sex, age, total amount of debt, and history of gambling were not significantly different, abstinent gamblers who had remained abstinent without relapse for at least three years experienced significantly more gambling symptoms (*P*=.01) than nonabstinent gamblers (Table 1). Although their gambling symptoms such as gambling tolerance, unsuccessful control over gambling, and reliance on others to provide money were not significantly different, abstinent gamblers had significantly higher preoccupation (*P*<.001) with gambling and lies for gambling compared with nonabstinent gamblers. The nonabstinent gamblers were significantly more experienced in illegal acts of gambling (*P*<.001) than abstinent gamblers. These findings indicated that abstinent gamblers experienced more gambling symptoms compared with nonabstinent gamblers although the effect size was small (*d*=0.179-0.261).

### Comparison of the Utterances in Web-Based Anonymous Gambler Chat Meetings Between Abstinent and Nonabstinent Gamblers

**Figure 4:**
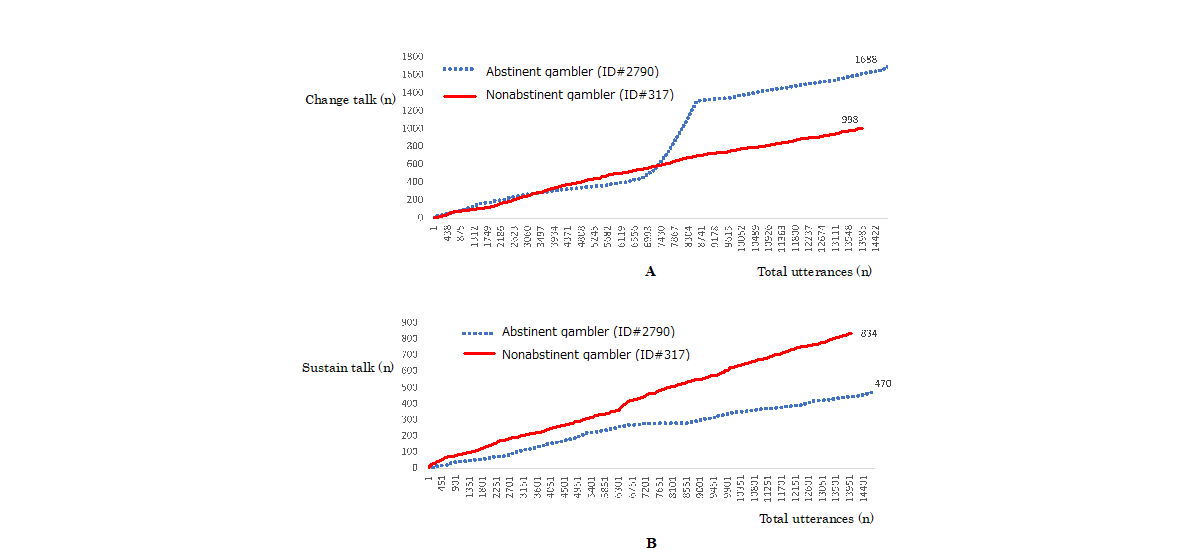
Dynamic differences in the change and sustain talks between abstinent and nonabstinent gamblers in web-based anonymous gambler chat meetings. Blue and red lines in A and B indicate abstinent (ID# 2790) and nonabstinent (ID# 317) gamblers, respectively. A: The accumulated number of change talks during web-based anonymous gambler chat meetings. The vertical and horizontal lines indicate the number of change talks and total utterances, respectively. B: The accumulated number of sustain talks during web-based anonymous gambler chat meetings. The vertical and horizontal lines indicate the number of sustain talks and total utterances, respectively.

Abstinent gamblers participated significantly longer in the meetings and talked more than nonabstinent gamblers (Table 1, both *P*<.001, *d*=0.590 and *d*=0.462, respectively). The average probability of change talks was not significantly different between abstinent and nonabstinent gamblers; however, the average probability of sustain talks in nonabstinent gamblers was significantly higher than that in abstinent gamblers (Table 1, *P*<.001, *d*=–0.369). Hence, the proportion of change talks’ probabilities (average probability of change talk/average probabilities of change and sustain talks) in abstinent gamblers was significantly higher than that in nonabstinent gamblers (Table 1, *P*<.001, *d*=0.285). Similarly, the proportion of change talks (number of change talks/number of change and sustain talks) in abstinent gamblers was significantly higher than that in nonabstinent gamblers (Table 1, *P*<.001, *d*=0.280). These findings indicate that abstinent gamblers showed higher proportion of change talks in the meetings compared with nonabstinent gamblers. To clarify the proportion of change talks between abstinent and nonabstinent gamblers, dynamic differences in the utterances between abstinent and nonabstinent gamblers were compared with similar number of utterances. Figure 4A shows the accumulated numbers of change talks between abstinent (ID# 2790) and nonabstinent (ID# 317) gamblers. Figure 4A indicates that abstinent gamblers produced more change talks than nonabstinent gamblers. Figure 4B shows the accumulated numbers of sustain talks between them. In contrast with that seen in change talks, nonabstinent gamblers produced more sustain talks than abstinent gamblers. Figure 4 shows that the utterances of abstinent and nonabstinent gamblers in the meetings showed dynamic differences.

### Development of the Change Talk Model for Abstinence

To visualize the dynamic differences between abstinent and nonabstinent gamblers, the change talk model for abstinence, involving the number of change and sustain talks as independent variables on x and y axes, respectively, classified 267 abstinent gamblers and 1625 nonabstinent gamblers (Figure 1A). Before classification, the correlations among the number of change talks, the number of sustain talks, and gambling abstinence were checked. The number of change and sustain talks were positively correlated with gambling abstinence (*r*=0.247, *r*=0.182, both were *P*<.001, n=1892). Moreover, the number of change talks was positively correlated with the number of sustain talks (*r*=0.676, *P*<.001, n=1892). These correlations indicated the positive relation of these talks with gambling abstinence.

Figure 1A shows abstinent and nonabstinent gamblers scattered on a map with the number of change and sustain talks on x and y axes, respectively. The abstinence likelihoods of gambling were also color-coded based on the SVM with RBF (accuracy was 0.8555, where cost of penalty and complexity of boundary area were 10^1^ and 10^–1^, respectively). The strong red color indicates low abstinence likelihood (negative evaluation scores) whereas the strong blue color indicates high abstinence likelihood (positive evaluation scores). Figure 1A shows that participants who increased the number of change and sustain talks were likely to move from the red zone to the blue zone, which indicated that they increased their abstinence likelihood of gambling.

### Clinical Use of the Change Talk Model for Abstinence

To clarify the clinical use of our model, 1 beginner and 2 intermediate gamblers were plotted, and the individual evaluation values for abstinence as well as their ideal proportion of change talks were shown through the gradient descent method (Figure 1B). To clarify the meanings of the number of change and sustain talks, the numbers were unstandardized. The beginner gambler (ID# 2665) started at 1 change talk and 1 sustain talk with evaluation value –1.000 and finished at 1657.16 change talks and 351.31 sustain talks with an evaluation value of –0.588 after 500 trials. The beginner gambler’s (ID# 2665) evaluation value at the end remained negative, thereby indicating low abstinence likelihood. The ideal portion of change talks (number of change talks/number of change and sustain talks) for this gamble during the web-based anonymous gambler chat meetings was regarded as 0.8254. One intermediate gambler (ID# 2162) started at 2130 change talks and 441 sustain talks with evaluation value –0.1167 and finished at 3731.94 change talks and 813.04 sustain talks with evaluation value 2.507 after 500 trials. His evaluation value became positive at the end, thereby indicating high abstinence likelihood. The best portion of change talks for him during the meeting was considered 0.8115. The other intermediate gambler (ID# 1008) started at 3396 change talks and 259 sustain talks with evaluation value –0.063 and finished at 4290.42 change talks and 787.67 sustain talks with evaluation value +2.815 after 500 trials. This gambler’s evaluation value turned positive at the end, thereby indicating high abstinence likelihood. The best portion of change talks for this gambler during the meetings was considered 0.6285. These findings indicate that the evaluation values are personalized feedback for gamblers. Furthermore, high proportions of change talks (over 0.80) were required for most beginner gamblers, whereas the best proportions of change talks were different among intermediate gamblers.

## Discussion

### Principal Results

The change talk classifier used in this study indicated 93% precision and analyzed 1.63 million utterances in web-based anonymous gambler chat meetings (Figure 3), thus leading to the development of the change talk model for gambling abstinence. The abstinent map with evaluation values showed the abstinence likelihoods of each gambler (Figure 1). Based on suggestions from a previous study on anonymous group meetings [4,5], this study confirmed that the abstinence process in web-based anonymous gambler chat meetings was similar to the process in other standardized therapies. Consistent with the results of previous studies regarding face-to-face motivational interviewing for gamblers [16,18,22,23], the proportion of change talks was also linked with improved outcome. In particular, the proportions at the initial stages were positively linked with 3-year continued gambling abstinence. This study advances previous findings regarding language classifiers in mental health fields [42,43] in 2 ways. First, the classifier used in previous studies classified the onset of mental disorders, whereas the classifier in this study classified recovery from a mental disorder. Second, the classifiers used in previous studies served as a screening tool for the early detection of mental disorders, whereas the classifier in this study is a therapeutic tool for identifying the recovery process. The 2D map with the abstinence likelihood slope used in this study was helpful for showing personalized evaluation values and ideal proportions of change talks, responding to every gambler’s utterance in the meetings.

This study involves 2 new methodologies: a change talk classifier and long-term data with dropout gamblers. First, this study used machine-learning methods and automatically classified gamblers’ utterances (Figure 3) [35]. Studies have utilized human expert resources to classify gamblers’ utterances [12,15,18-20,44-47], with limited data size (around 10^3^) and generalization of their findings. The change talk classifier used in this study increased data size (over 10^6^) similar to recent mental health research [8,42,43] and was applicable to other therapy processes focusing on change talks such as group and individual therapies for healthy dieting and prevention of substance abuse [16,23]. The utilization of the change talk classifier for these therapies can save human resources about change talk classification [12,19,20] and broaden the applicability of change talk classifications or the core scheme of motivational interviewing [14-16] for addictive disorders [7,21].

This study also involved long-term data (maximum participation length of 3872 days) with both dropout and ongoing data sets (Figure 2, Table 1). Inclusion of these data sets enabled extensive and detailed slope maps of abstinence likelihoods of gambling. Studies have primarily utilized the initial and final sessions’ scores of gamblers who completed the treatments. However, considering their high dropout rates [48,49], these data sets could bias gamblers’ characteristics. Moreover, the data set involving only the first and last sessions does not show the processes in between, and the model based on these data sets does not reveal the abstinence likelihood corresponding to the therapy processes [50]. Long-term data with dropout and ongoing data sets can be helpful for estimating changes during therapy, particularly for diseases that require extensive treatment periods [25-28].

### Limitations

This study has 5 limitations. First, the number of female participants in the study sample was limited. The abstinence process differs between female and male participants [51]; therefore, future studies must involve more female participants. Second, the current measurement of gamblers’ symptoms was based on their utterances in web-based anonymous gambler chat meetings. Hence, our measurement may underestimate several gambling symptoms because the measurement could not assess these symptoms that gamblers did not reveal in the meetings. To compensate for these limitations, questionnaires [32] or standardized interview [31] data are required in future studies. Third, this study identified gamblers through their anonymous names, but it is possible that 2 different gamblers used the same anonymous name or that one gambler used multiple anonymous names. Their identities could not be checked because of the web-based anonymity settings. Another data set such as physical meetings [24,49] would be helpful for validating the findings of this study. Fourth, the small number of long-term participants is also one of the study limitations. This study model shows the highest likelihood area for gambling abstinence (Figure 1A, top right most blue area); this implies that individuals in this area will not benefit from participating in web-based anonymous gambler chat meetings. This point must be re-examined by collecting more data regarding long-term participants. Fifth, the internal validity of this study was limited because abstinent and nonabstinent gamblers freely interacted and the proportion of their change talks was not manipulated. To increase the internal validity, future studies must consider an experimental design.

### Conclusion

Despite these limitations, this study developed the change talk model for gambling abstinence in web-based anonymous gambler chat meetings with the change talk classifier. The change talk classifier used in this study increased the data size of psychotherapies [6,12,13,18,44-47] and broadened the applicability of language analysis on social media for mental health services [42,43]. The change talk model for abstinence used in this study can provide personalized evaluation scores for abstinence and an ideal proportion of change talks to the gamblers responding to every utterance during the meetings. In clinical settings, using this model, participants can determine their abstinence likelihood because the evaluation values are determined based on their previous utterances before they participate in the meetings (Figure 1B). The ideal proportion of change talks can be estimated using this model (Figure 1B) and they can be motivated to comment at the meetings based on that proportion. After the meeting, gamblers can also observe changes in their evaluation values during the meeting, thus allowing them to objectively evaluate their individual progress of each meeting (Figure 1B). These factors are considered to provide personalized feedback for participants [14,17] and can likely lead to improved outcomes [38]. Providing personalized evaluation values and the optimal proportion of change talks, which were also validated in millions of data sets, can increase the abstinence likelihood of gamblers. This may help to prevent severe mental, social, and financial problems caused by the gambling disorder [1-3].

## Appendix

Multimedia Appendix 1 Supplementary data.

## Abbreviations

RBF: radial basis kernel function
SVM: support vector machine

## References

[1] (2013). Diagnostic and Statistical Manual of Mental Disorders (DSM-5).

[2] McLellan A, Lewis D, O'Brien C P, Kleber H (2000). Drug dependence, a chronic medical illness: implications for treatment, insurance, and outcomes evaluation. JAMA.

[3] Project MATCH Research Group (1998). Matching alcoholism treatments to client heterogeneity: Project MATCH three-year drinking outcomes. Alcohol Clin Exp Res.

[4] Kelly J, Hoeppner B, Stout R, Pagano M (2012). Determining the relative importance of the mechanisms of behavior change within Alcoholics Anonymous: a multiple mediator analysis. Addiction.

[5] Kelly J (2017). Is Alcoholics Anonymous religious, spiritual, neither? Findings from 25 years of mechanisms of behavior change research. Addiction.

[6] Mujcic A, Linke S, Hamilton F, Phillips A, Khadjesari Z (2020). Engagement With Motivational Interviewing and Cognitive Behavioral Therapy Components of a Web-Based Alcohol Intervention, Elicitation of Change Talk and Sustain Talk, and Impact on Drinking Outcomes: Secondary Data Analysis. J Med Internet Res.

[7] D'Amico EJ, Houck JM, Hunter SB, Miles JNV, Osilla KC, Ewing BA (2015). Group motivational interviewing for adolescents: change talk and alcohol and marijuana outcomes. J Consult Clin Psychol.

[8] Xiao B, Can D, Gibson J, Imel Z, Atkins D, Georgiou P, Narayanan S (2016). Behavioral coding of therapist language in addiction counseling using recurrent neural networks.

[9] Challet-Bouju G, Hardouin J, Thiabaud E, Saillard A, Donnio Y, Grall-Bronnec M, Perrot B (2020). Modeling Early Gambling Behavior Using Indicators from Online Lottery Gambling Tracking Data: Longitudinal Analysis. J Med Internet Res.

[10] Rodda S, Lubman DI, Dowling NA, Bough A, Jackson AC (2013). Web-based counseling for problem gambling: exploring motivations and recommendations. J Med Internet Res.

[11] Bem D (1972). Self-Perception Theory. Advances in Experimental Social Psychology.

[12] Amrhein PC, Miller WR, Yahne CE, Palmer M, Fulcher L (2003). Client commitment language during motivational interviewing predicts drug use outcomes. Journal of Consulting and Clinical Psychology.

[13] Miller W, Benefield R, Tonigan J (1993). Enhancing motivation for change in problem drinking: A controlled comparison of two therapist styles. Journal of Consulting and Clinical Psychology.

[14] Miller W, Rollnick S (2012). Motivational Interviewing: Helping People Change.

[15] Martin T, Moyers T, Houck J, Christopher P, Miller W (2005). Motivational Interviewing Sequential Code for Observing Process Exchanges (MI-SCOPE) Coder's Manual.

[16] Magill M, Apodaca TR, Borsari B, Gaume J, Hoadley A, Gordon REF, Tonigan JS, Moyers T (2018). A meta-analysis of motivational interviewing process: Technical, relational, and conditional process models of change. J Consult Clin Psychol.

[17] Miller WR, Rose GS (2009). Toward a theory of motivational interviewing. Am Psychol.

[18] Hodgins DC, Ching LE, McEwen J (2009). Strength of commitment language in motivational interviewing and gambling outcomes. Psychol Addict Behav.

[19] Houck J, Moyers T, Miller W, Glynn L, Hallgren K (2010). Motivational interviewing skill code (MISC) 2.5. ELICIT Coding Manual.

[20] Glynn L, Moyers T (2012). Manual for the client language easy rating (clear) coding system: formerly “Motivational Interviewing Skill Code (MISC) 1.1”. Center on Alcoholism, Substance Abuse, and Addictions.

[21] Romano M, Peters L (2015). Evaluating the mechanisms of change in motivational interviewing in the treatment of mental health problems: A review and meta-analysis. Clin Psychol Rev.

[22] Swan J (2019). The technical hypothesis of motivational interviewing: an examination of change language in traditional and computer-based MI for disordered gamblers. University of Calgary Library Resources.

[23] Magill M, Gaume J, Apodaca TR, Walthers J, Mastroleo NR, Borsari B, Longabaugh R (2014). The technical hypothesis of motivational interviewing: a meta-analysis of MI's key causal model. J Consult Clin Psychol.

[24] Joramo I, Solem S, Romundstad B, Nordahl HM (2021). Change talk and sustain talk in treatment of generalized anxiety disorder: A secondary analysis of cognitive behavioral therapy and metacognitive therapy in adult outpatients. J Behav Ther Exp Psychiatry.

[25] Richmond RL, Kehoe L, de Almeida Neto A C (1997). Three year continuous abstinence in a smoking cessation study using the nicotine transdermal patch. Heart.

[26] Silverman K, Svikis D, Wong CJ, Hampton J, Stitzer ML, Bigelow GE (2002). A reinforcement-based Therapeutic Workplace for the treatment of drug abuse: Three-year abstinence outcomes. Experimental and Clinical Psychopharmacology.

[27] Slutske W (2006). Natural Recovery and Treatment-Seeking in Pathological Gambling: Results of Two U.S. National Surveys. American Journal of Psychiatry.

[28] Oei TPS, Gordon LM (2008). Psychosocial factors related to gambling abstinence and relapse in members of gamblers anonymous. J Gambl Stud.

[29] Okui T (2008). Bulletin board for overcoming gambling addiction-No cracks, no slots, no gambling!. Support Addict Gamblers.

[30] McGrath Daniel S, Barrett S (2009). The comorbidity of tobacco smoking and gambling: a review of the literature. Drug Alcohol Rev.

[31] Grant JE, Steinberg MA, Kim SW, Rounsaville BJ, Potenza MN (2004). Preliminary validity and reliability testing of a structured clinical interview for pathological gambling. Psychiatry Res.

[32] Yokotani K, Tamura K, Kaneko Y, Kamimura E (2020). Craving for Gambling Predicts Income-Generating Offenses: A Pathways Model of a Japanese Prison Population. J Gambl Stud.

[33] Landis JR, Koch GG (1977). The Measurement of Observer Agreement for Categorical Data. Biometrics.

[34] NTT Communications A natural language processing and speech processing API platform developed by NTT Communications that utilizes one of the largest Japanese language dictionaries in Japan. COTOHA API.

[35] Mekala D, Gupta V, Paranjape B, Karnick H (2017). SCDV: Sparse Composite Document Vectors using soft clustering over distributional representations.

[36] Rondhuit.

[37] Murata M, Isahara H, Nagao M Resolution of indirect anaphora in Japanese sentences using examples "X no Y (Y of X)". ACL Anthology.

[38] Yokotani K, Tamura K (2015). Effects of Personalized Feedback Interventions on Drug-Related Reoffending: a Pilot Study. Prev Sci.

[39] Hsu C, Chang C, Lin C A practical guide to support vector classification. National Taiwan University Repository.

[40] Faul F, Erdfelder E, Buchner A, Lang A (2009). Statistical power analyses using G*Power 3.1: Tests for correlation and regression analyses. Behavior Research Methods.

[41] Kudo T (2006). MeCab: Yet Another Part-of-Speech and Morphological Analyzer.

[42] Choudhury M, Gamon M, Counts S, Horvitz E Predicting depression via social media. ICWSM.

[43] Reece AG, Reagan AJ, Lix KLM, Dodds PS, Danforth CM, Langer EJ (2017). Forecasting the onset and course of mental illness with Twitter data. Sci Rep.

[44] Baer J, Beadnell B, Garrett S, Hartzler B, Wells E, Peterson P (2008). Adolescent change language within a brief motivational intervention and substance use outcomes. Psychol Addict Behav.

[45] Bertholet N, Faouzi M, Gmel G, Gaume J, Daeppen Jean-Bernard (2010). Change talk sequence during brief motivational intervention, towards or away from drinking. Addiction.

[46] Hodgins D, Currie S, Currie G, Fick G (2009). Randomized trial of brief motivational treatments for pathological gamblers: More is not necessarily better. J Consult Clin Psychol.

[47] Vader A, Walters S, Prabhu G, Houck J, Field C (2010). The language of motivational interviewing and feedback: counselor language, client language, and client drinking outcomes. Psychol Addict Behav.

[48] Melville KM, Casey LM, Kavanagh DJ (2007). Psychological treatment dropout among pathological gamblers. Clin Psychol Rev.

[49] Pallesen S, Mitsem M, Kvale G, Johnsen B, Molde H (2005). Outcome of psychological treatments of pathological gambling: a review and meta-analysis. Addiction.

[50] Russ TC, Woelbert E, Davis KAS, Hafferty JD, Ibrahim Z, Inkster B, John A, Lee W, Maxwell M, McIntosh AM, Stewart R, MQ Data Science group (2019). How data science can advance mental health research. Nat Hum Behav.

[51] Slutske W, Blaszczynski A, Martin N (2009). Sex differences in the rates of recovery, treatment-seeking, and natural recovery in pathological gambling: results from an Australian community-based twin survey. Twin Res Hum Genet.

