# Supplementary material for: A Change Talk Model for Abstinence Based on Web-Based Anonymous Gambler Chat Meeting Data by Using an Automatic Change Talk Classifier: Development Study
Source: J Med Internet Res. 2021 Jun 21;23(6):e24088. doi: 10.2196/24088 (PMC8277414; doi:10.2196/24088)
Supplement: Multimedia Appendix 1 [file jmir_v23i6e24088_app1.docx]

**Table S1.** Concordance rates of gambling symptoms between experts and raters.

| Characteristics | Rater A | Rater B | Criterion |
| --- | --- | --- | --- |
| No gambling symptoms | 0.835 | 0.860 | ** |
| Gambling Tolerance | 0.730 | 0.797 | * |
| Gambling Withdrawal | 0.529 | 0.508 |  |
| Unsuccessful control over gambling | 0.799 | 0.818 | * |
| Preoccupied with gambling | 0.667 | 0.726 | * |
| Gambling as problem avoidance | 0.586 | 0.656 |  |
| Chasing one’s gambling loss | 0.735 | 0.138 |  |
| Lies for gambling | 0.805 | 0.777 | * |
| Loss of relationships and opportunities | 0.492 | 0.451 |  |
| Reliance on others to provide money | 0.651 | 0.884 | * |
| Illegal acts for gambling | 0.658 | 0.795 | * |

*Notes:* All scores indicate Kappa coefficients.

**: The agreements between the expert and the two raters were over 0.80 (almost perfect agreement).

*: The agreements between the expert and the two raters were over 0.60 (substantial agreement).
